# Association between adenovirus viral load and mortality in pediatric allo-HCT recipients: the multinational AdVance study

**DOI:** 10.1038/s41409-019-0483-7

**Published:** 2019-02-25

**Authors:** Marco Zecca, Robert Wynn, Jean-Hugues Dalle, Tobias Feuchtinger, Enrikas Vainorius, Thomas M. Brundage, Aastha Chandak, Essy Mozaffari, Garrett Nichols, Franco Locatelli

**Affiliations:** 1 Pediatric Hematology/Oncology, Fondazione IRCCS Policlinico S. Matteo, Pavia, Italy; 2 Royal Manchester Children’s Hospital, Manchester, UK; 3 Robert Debré Hospital, Assistance Publique-Hôpitaux de Paris and Paris Diderot University, Paris, France; 4 Department of Pediatric Hematology/Oncology and Stem Cell Transplantation, Dr. von Hauner University Children’s Hospital LMU Munich, Munich, Germany; 5 Chimerix, Durham, NC USA; 6 Analytica Laser, New York, NY USA; 7 Department of Pediatric Hematology/Oncology, IRCCS, Ospedale Pediatrico Bambino Gesù, University La Sapienza, Rome, Italy

**Keywords:** Infectious diseases, Risk factors

## Abstract

This multivariable analysis from the AdVance multicenter observational study assessed adenovirus (AdV) viremia peak, duration, and overall AdV viral burden—measured as time-averaged area under the viremia curve over 16 weeks (AAUC_0-16_)—as predictors of all-cause mortality in pediatric allo-HCT recipients with AdV viremia. In the 6 months following allo-HCT, 241 patients had AdV viremia ≥ 1000 copies/ml. Among these, 18% (43/241) died within 6 months of first AdV ≥ 1000 copies/ml. Measures of AdV viral peak, duration, and overall burden of infection consistently correlate with all-cause mortality. In multivariable analyses, controlling for lymphocyte recovery, patients with AdV AAUC_0-16_ in the highest quartile had a hazard ratio of 11.1 versus the lowest quartile (confidence interval 5.3–23.6); for peak AdV viremia, the hazard ratio was 2.2 for the highest versus lowest quartile. Both the peak level and duration of AdV viremia were correlated with short-term mortality, independent of other known risk factors for AdV-related mortality, such as lymphocyte recovery. AdV AAUC_0-16_, which assesses both peak and duration of AdV viremia, is highly correlated with mortality under the current standard of care. New therapeutic agents that decrease AdV AAUC_0-16_ have the potential of reducing mortality in this at-risk patient population.

## Introduction

Pediatric allogeneic hematopoietic stem cell transplant (allo-HCT) recipients with nonmatched sibling sources are at high risk of developing adenovirus (AdV) infections, particularly during the immediate posttransplant period before immune reconstitution has occurred [1–7]. AdV viremia is widely considered to be an indicator of disease dissemination [6, 8, 9], with higher levels of AdV viremia (≥1000 copies/ml) shown to be associated with mortality in single-center studies [7, 10]. Guidelines have thus recommended antiviral treatment in high-risk patients with AdV viremia [11].

While there are few treatment options for AdV infection in allo-HCT recipients, the Infectious Diseases Working Party of the European Society of Blood and Marrow Transplantation recommends, whenever feasible, tapering immunosuppression and considering off-label treatment with intravenous (IV) cidofovir when AdV viremia is documented [7]. However, data supporting efficacy of IV cidofovir are limited, and treatment responses are generally observed only when immune reconstitution has occurred [11, 12]. The use of IV cidofovir is also associated with nephrotoxicity, particularly in younger children [13]. Until a more effective or less toxic therapy is available, however, treatment with IV cidofovir is likely to be considered for allo-HCT recipients with confirmed AdV viremia.

Although there is accumulating evidence of an association between AdV viremia and mortality, these data come from small or single-center studies, thus raising questions as to how generalizable the findings may be for other centers, settings, or countries [1, 14, 15]. There is therefore a critical need for robust data from multinational and multicenter studies evaluating the relationship between AdV viral burden and mortality in pediatric allo-HCT recipients under the current standard of care in order to better define the baseline upon which therapeutic improvements may be built.

In order to understand the relationship between AdV viremia and outcomes, it is important to identify the viral dynamic parameters that best define the course of AdV viremia. It is known that the median time to AdV clearance with treatment with cidofovir is 9 weeks (range, 6–15 weeks) [12]. Previous studies have also shown that peak AdV viremia levels are independently associated with mortality; [16] the impact of persistent viremia, even at lower levels, could indicate ongoing organ damage for this lytic viral disease [17]. Other parameters, such as time-averaged AdV area under the curve (AAUC), that incorporate both peak and persistence of AdV viremia could also correlate with clinical outcomes.

We investigated the association of AdV viral dynamics with all-cause mortality in pediatric patients with AdV viremia ≥ 1000 copies/ml using data from the AdVance study, a multicenter retrospective study of AdV infection in allo-HCT recipients under current management [11, 18].

## Methods

### Study design and participants

AdVance was a retrospective, multicenter, multinational study investigating the incidence, management, and clinical outcomes of AdV infection in 2540 adult and 1736 pediatric allo-HCT recipients. There were 50 participating centers from seven European countries, including the United Kingdom, France, Germany, Italy, Spain, the Netherlands, and the Czech Republic. Of the 50 participating centers, at least 37 treated one or more pediatric patients. The study captured data from allogeneic transplants performed at the participating centers between January 2013 and September 2015, during a timeframe preceding the availability of brincidofovir at each center, and included follow-up data from all subjects until death or 1 year posttransplant. This analysis focuses on pediatric (<18 years) allo-HCT recipients who had AdV viremia (measured either in plasma or whole blood) ≥ 1000 copies/ml within the first 6 months post-HCT.

Data were extracted by qualified and trained personnel at each study center from medical records and case report forms for all pediatric allo-HCT recipients who met inclusion criteria. The study was approved by institutional review boards/ethics committees at each participating center and data collection and use were in accordance with local and national laws. Extracted data included demographics, underlying disease for which allo-HCT was performed, details of the stem cell source used and type of donor, use of ex vivo T-cell depletion or serotherapy, and presence of comorbidities (Supplementary Table 1). Lowest lymphocyte count and highest serum creatinine level within 30-day windows were captured, as were highest organ stage of graft-versus-host disease (GvHD) at time of AdV viremia ≥ 1000 copies/ml and use of renal replacement (hemodialysis, peritoneal dialysis, ultrafiltration, and continuous venovenous filtration therapy). The results of all AdV tests performed were captured, as were antivirals used. The primary outcome measure was mortality within 6 months of first AdV viremia ≥ 1000 copies/ml.

### Statistical methods

**Fig. 1:**
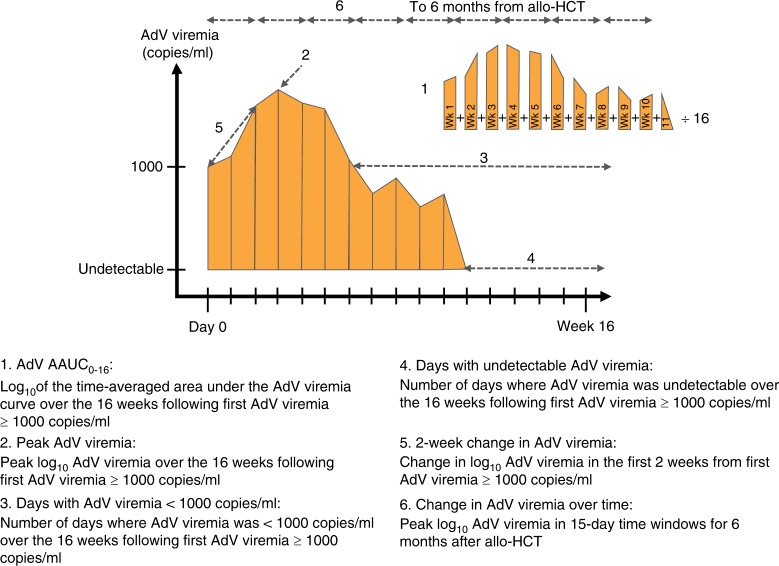
AdV viral load dynamics

Potential predictors of mortality were included in univariate and multivariable modeling. Factors with a *P*-value ≤ 0.20 in univariate analysis were assessed in the multivariable models with backward selection used to build the final model with an exit criterion of *P*-value ≤ 0.10. Separate multivariable models were analyzed, each including one of six AdV virologic measures (Fig. 1). Data were censored at date of last follow-up, or date of death, if these events occurred before 16 weeks from first detection of AdV viremia ≥ 1000 copies/ml; separate models were generated for overall mortality and mortality that occurred in the absence of relapsed disease. The virologic measures evaluated were:

1. Time-averaged AUC for AdV viremia from first detection of AdV viremia ≥ 1000 copies/ml for 16 weeks (AAUC_0-16_).
2. Peak viremia: defined as the peak level of AdV viremia attained within 16 weeks from first detection of AdV viremia ≥ 1000 copies/ml.
3. Days with viremia < 1000 copies/ml within 16 weeks after first detection of AdV viremia ≥ 1000 copies/ml.
4. Days with undetectable viremia within 16 weeks after first detection of AdV viremia ≥ 1000 copies/ml.
5. Two-week change in AdV viremia: defined as change at 2 weeks from first detection of AdV viremia ≥ 1000 copies/ml. This change (positive or negative) is calculated by subtracting AdV viremia at first detection ≥ 1000 copies/ml from AdV viremia at 2 weeks.
6. Change in AdV viremia over time: peak AdV viremia as a time-varying covariate for the hazard of mortality for each patient in 15-day windows from Day 0 to Day 180 or until death or date of last follow-up.

In addition to the virologic measures noted above, the following clinical and demographic factors were assessed as potential covariates in each of the models:

1. Immune reconstitution measured as total lymphocyte count categories of <300, 300–899, ≥900 cells/µl in 30-day time-dependent windows from Day 0 to Day 180 or until death or date of last follow-up.
2. Use of cidofovir, defined as time-varying covariate from the date of first use.
3. Maximum GvHD stage across skin, liver, and gastrointestinal tract at the time of first detection of AdV viremia ≥ 1000 copies/ml (stage 0, stage 1 or 2, stage 3 or 4).
4. Use of renal replacement therapy as time-varying covariate from the date of first use.
5. Other baseline factors included gender, underlying disease, age at the time of transplant, type of donor, T-cell depletion/serotherapy, comorbidities (Supplementary Table 1), diagnosis of AdV disease (possible/probable, definitive, or disseminated as per study protocol definition), time from allo-HCT to AdV infection, time from AdV infection to AdV viremia ≥ 1000 copies/ml, time from allo-HCT to AdV viremia ≥ 1000 copies/ml, and presence of other double-stranded DNA (dsDNA) viral coinfections (cytomegalovirus, Epstein-Barr virus, or BK virus), as defined in Table 1.

**Table 1:** Variables included in multivariable analysis models

Prognostic factors	Definition
Lymphocyte count over time	<300, 300–899, ≥900 30-day time-dependent windows post first detection of AdV viremia ≥ 1000 copies/ml
CDV use over time	Time-dependent covariate from time of treatment initiation
Acute GvHD (maximum grading across organs)	0, 1–2, 3–4
Use of renal replacement therapy over time	Time-dependent covariate from time of treatment initiation
Country	UK, Spain, France, Italy, Germany, Netherlands, Czech Republic
Gender	Male, female
Underlying disease	Malignant, nonmalignant immunodeficient, nonmalignant immunocompetent
Age at time of transplant	<2, 2– < 12, 12– < 18 years
Type of donor	MRD or MUD, MMD, haploidentical or cord blood
T-cell depletion/serotherapy	None, Alemtuzumab (Campath), ATG, ex vivo T-cell depletion
Comorbidities	None, ≥1
AdV disease	No, yes
Time from allo-HCT to AdV infection	Weeks, continuous
Time from AdV infection to AdV viremia ≥ 1000 copies/ml	Weeks, continuous
Time from allo-HCT to AdV viremia ≥ 1000 copies/ml	Weeks, continuous
Coinfections (dsDNA viral infections: CMV, EBV, BKV)	None, ≥1 dsDNA virus in addition to AdV

Malignant: acute lymphoblastic leukemia, acute myeloid leukemia, chronic myelogenous leukemia, myelodysplasia, non-Hodgkin lymphoma, other malignancy. Nonmalignant immunodeficient: autoimmune disease, congenital immunodeficiency, other congenital. Nonmalignant immunocompetent: aplastic anemia, sickle cell anemia, thalassemia

*ATG* antithymocyte globulin, *BKV* BK virus, *CDV* cidofovir, *CMV* cytomegalovirus, *EBV* Epstein-Barr virus, *MMD* mismatched donor, *MRD* matched related donor, *MUD* matched unrelated donor

Cox proportional hazards models were used to examine time to all-cause mortality within 6 months of first AdV viremia ≥ 1000 copies/ml. Fine-Gray models were used to examine time to nonrelapse-related mortality within 6 months of first AdV viremia ≥ 1000 copies/ml, with relapse considered as a competing risk.

Quartile analyses were conducted to examine the association between all the virologic measures (with the exception of time-varying AdV viremia), and time to all-cause and nonrelapse-related mortality. Patients were divided into quartiles according to the values of each of the continuous virologic measures, and the median values in each quartile were identified. The hazard ratios of the virologic measures (when used as a continuous variable) obtained from the final multivariable models were then used to calculate the hazard ratios for the comparison between the median values of the highest quartile versus the lowest quartile.

A Kaplan–Meier curve was generated for the time from first detection of AdV viremia ≥ 1000 copies/ml to all-cause mortality. Patients were censored at 6 months or at the date of last contact (if lost to follow-up before 6 months); the patients were stratified by quartiles of AdV AAUC_0-16_. For nonrelapse-related mortality, a cumulative incidence curve for time from first detection of AdV viremia ≥ 1000 copies/ml to nonrelapse-related mortality was generated. Relapse was considered as a competing risk, and patients were censored at 6 months or at the date of last contact or at the date of relapse (if lost to follow-up or relapsed before 6 months).

## Results

AdVance included 1736 pediatric allo-HCT recipients transplanted during the study period. Of these, 32% (558/1736) had at least one positive AdV test within 6 months of allo-HCT and 23% (395/1736) had AdV viremia. This analysis was based on the 241 pediatric allo-HCT recipients who had AdV viremia ≥ 1000 copies/ml (Table 2). The median age was 5 years, with a range of less than 1 to 17 years. The majority of patients were male (66%), and matched unrelated transplants (42%) were the most frequent. Most patients (87%) had undergone ex vivo T-cell depletion or received serotherapy (antithymocyte globulin [ATG] or alemtuzumab). At the time of first detection of AdV viremia ≥ 1000 copies/ml, 80% of patients had lymphocyte counts < 300 cells/µl. Concurrent dsDNA viral infections were relatively common, with 43% of patients having at least one additional dsDNA viral infection at the time of first AdV infection detection. In these pediatric allo-HCT recipients who developed AdV viremia ≥ 1000 copies/ml within 6 months after transplant, all-cause mortality was 18% (43/241; 95% confidence interval [CI] 13.2–22.4), and nonrelapse-related mortality was 15% (35/241; 95% CI 10.3–18.8).

**Table 2:** Demographics and baseline characteristics

Baseline characteristics	Pediatric patients (*N* = 241)
*Age, years*	
Mean (s.d.)	6.3 (4.9)
Median (range)	5.0 (<1.0–17.0)
*Age stratification, years*, *n* (%)	
<2	61 (25.3)
2 to <6	63 (26.1)
6 to <12	72 (29.9)
12 to <18	45 (18.7)
*Gender*, *n* (%)	
Male	159 (66.0)
Female	82 (34.0)
*Stem cell source*, *n* (%)	
BM	116 (48.1)
PBSC	92 (38.2)
Cord blood unit(s)	33 (13.7)
*Donor type*^a^, *n* (%)	
Matched unrelated	100 (41.5)
Haploidentical	49 (20.3)
Mismatched^b^	43 (17.8)
Cord blood unit(s)	35 (14.5)
Matched related	33 (13.7)
*T-cell depletion*^c^, *n* (%)	
ATG	95 (39.4)
Alemtuzumab (Campath)	62 (25.7)
Ex vivo	53 (22.0)
None	31 (12.9)
*Time from AdV infection to AdV* *≥* *1000 copies/ml, days*	
Mean (s.d.)	15.2 (24.5)
Median (range)	7 (0.0–146.0)
IQR	0.0–17.0
<28 days, *n* (%)	201 (83.4)
≥28 days, *n* (%)	40 (16.6)
*Time from allo-HCT to AdV* *≥* *1000 copies/ml, days*	
Mean (s.d.)	40.4 (38.8)
Median (range)	26.0 (0.0–174.0)
IQR	13.0–56.0
<28 days, *n* (%)	130 (53.9)
≥28 days, *n* (%)	111 (46.1)
*Disease for which allo-HCT was conducted*, *n* (%)	
Malignant	148 (61.4)
Nonmalignant immunodeficient	72 (29.9)
Nonmalignant immunocompetent	21 (8.7)
*dsDNA viral coinfections*, *n* (%)	
None	138 (57.3)
≥1 dsDNA viral infection in addition to AdV	103 (42.7)
*Comorbidities*^d^, *n* (%)	
None	147 (61.0)
≥1	94 (39.0)
*Lymphocyte count at time of AdV viremia* *≥* *1000 copies/ml*, *n* (%)	
≥900	14 (5.8)
300–899	35 (14.5)
<300	192 (79.7)
*Maximum stage GvHD among skin, liver, GI at the time of AdV viremia* *≥* *1000 copies/ml*, *n* (%)	
0	174 (72.2)
1	22 (9.1)
2	17 (7.1)
3	16 (6.6)
4	12 (5.0)

*ATG* antithymocyte globulin, *BM* bone marrow, *GI* gastrointestinal, *IQR* interquartile range, *PBSC* peripheral blood stem cell, *s.d.* standard deviation

^a^Categories are not mutually exclusive

^b^Mismatching was determined according to standard practice at each study site

^c^A hierarchy was applied such that patients who received ex vivo T-cell depletion and ATG and/or alemtuzumab were counted in the ex vivo category

^d^Listed in Supplementary Table 1

### Univariate and multivariable analyses

Each of the six AdV viral load measures was associated with 6-month all-cause mortality in univariate models (Table 3). Each log_10_ increase in peak AdV viral load was associated with a 47% increase in the hazard for mortality. The relationship between peak viral load and mortality was greatest at the top of the range—10% of pediatric patients achieved peak AdV viral loads greater than 4.75 × 10^6^ copies/ml, with correlating mortality of 58% (14/24 subjects); this was dramatically higher than the 13% (29/217 subjects) mortality observed in the remaining 90% of the AdVance pediatric population. For AdV AAUC_0-16_, each log_10_ increase in average viral burden was associated with a doubling of the hazard for mortality. Each day with AdV viremia < 1000 copies/ml and each day with AdV undetectable over the 16 weeks after AdV viremia ≥ 1000 copies/ml was associated with a 4% reduction in the hazard for mortality. In addition to the AdV viral load measures, the presence of AdV disease was also significantly associated with mortality. Time-updated lymphocyte counts, the use of IV cidofovir, maximum GvHD stage ≥ 3, the use of renal replacement therapy, the presence of comorbidities, and donor type were associated with increased mortality risk (Table 3).

**Table 3:** Univariate analysis

Prognostic factor	Definition	Avg. *N*	HR (95% CI)	Pairwise *P-*value	Overall *P-*value
AdV AAUC_0-16_	Continuous	241	1.99 (1.69–2.35)		<0.0001
Peak AdV viremia_0-16_	Continuous	241	1.47 (1.29–1.67)		<0.0001
Days < 1000 copies/ml_0-16_	Continuous	241	0.96 (0.95–0.97)		<0.0001
Days with undetectable viremia_0-16w_	Continuous	241	0.96 (0.95–0.97)		<0.0001
2-week change in AdV viremia	Continuous	241	1.35 (1.15–1.59)		0.0003
Change in AdV viremia over time	Continuous	241	1.54 (1.39–1.71)		<0.0001
Lymphocyte counts (t)	≥900	65.0			<0.0001
	300–899	76.0	1.91 (0.48–7.56)	0.3557	
	<300	77.3	10.95 (3.18–37.71)	0.0001	
CDV (t) use	No	121	1.00		0.0310
	Yes	120	1.98 (1.06–3.68)		
Maximum GvHD stage	0	174	1.00		0.0153
	1, 2	39	0.76 (0.29–1.97)	0.5738	
	3, 4	28	2.67 (1.30–5.51)	0.0076	
Renal replacement (t)	No	229	1.00		<0.0001
	Yes	12	28.41 (13.02–61.98)		
Gender	Male	159	1.00		0.1170
	Female	82	1.62 (0.89–2.95)		
Type of donor	MRD or MUD	124	1.00		0.0221
	MMD/Hapl/CBU	117	2.08 (1.11–3.89)		
Comorbidities^a^	None	147	1.00		0.0132
	≥1	94	2.14 (1.17–3.91)		
AdV disease	No	158	1.00		<0.0001
	Yes	83	2.94 (1.60–5.39)		

*CBU* cord blood (units), *CDV* cidofovir, *Hapl* haploidentical, *HR* hazard ratio, *MMD* mismatched donor, *MRD* matched related donor, *MUD* matched unrelated donor

^a^Listed in Supplementary Table 1

**Fig. 2:**
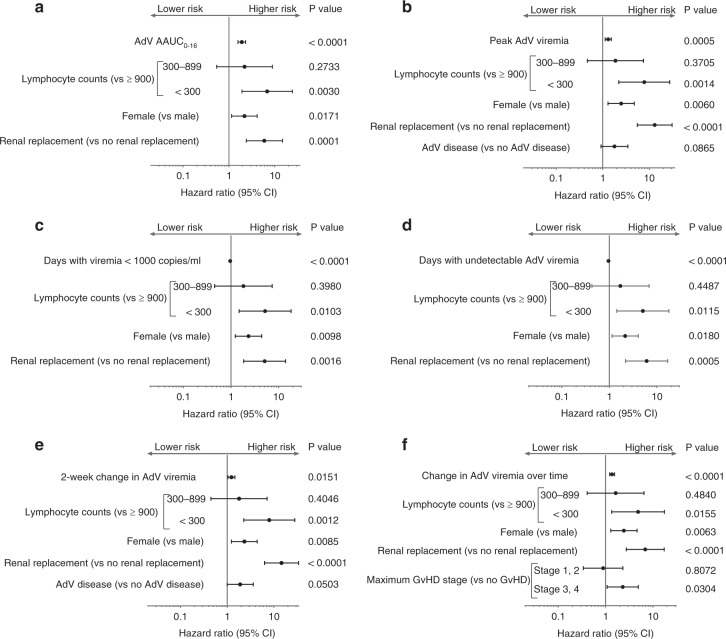
All six measures of AdV viral load dynamics were independently associated with increased risk of all-cause mortality. **a** AdV AAUC_0-16_. **b** Peak AdV viremia_0-16_. **c** Days with viremia < 1000 copies/ml_0-16_. **d** Days with undetectable viremia < 1000 copies/ml_0-16_. **e** 2-week change in AdV viremia. **f** Change in AdV viremia over time

In multivariable analysis, each of the aforementioned virologic measures was a strong predictor of mortality in separate models (Fig. 2). In addition, time-updated low lymphocyte counts (<300 cells/µl), female gender, and use of renal replacement therapy were also significant predictors (Fig. 2). In the model including maximum viremia over time, GvHD stage 3 or 4 was also a significant predictor of mortality (Fig. 2).

### Quartile analyses

**Fig. 3:**
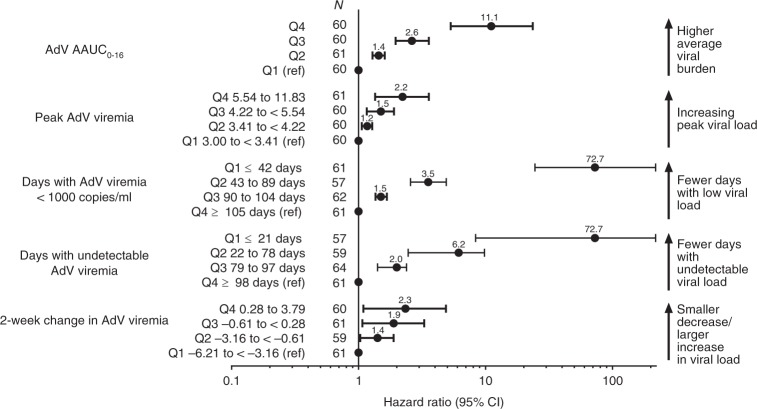
Higher AdV viral load and burden were associated with a significantly greater risk of all-cause mortality in multivariable models. AdV AAUC_0-16_, log_10_ of the time-averaged area under the AdV viremia curve over the 16 weeks following first AdV viremia ≥ 1000 copies/ml; Peak AdV viremia, peak log_10_ AdV viremia over the 16 weeks following first AdV viremia ≥ 1000 copies/ml; Days with AdV viremia < 1000 copies/ml, number of days where AdV viremia was <1000 copies/ml over the 16 weeks following first AdV viremia ≥ 1000 copies/ml; Days with undetectable AdV viremia, number of days where AdV viremia was undetectable over the 16 weeks following first AdV viremia ≥ 1000 copies/ml; 2-week change in AdV viremia, change in log_10_ AdV viremia in the first 2 weeks from first AdV viremia ≥ 1000 copies/ml. Q, quartile; ref, reference group. Note: Factors with *P* *≤* 0.20 in the univariate analysis were brought forward into the multivariable models, followed by a backward selection to keep the factors with *P* *≤* 0.10 in the final models. Lymphocyte count, gender, and renal replacement therapy were also significant (*P* *<* 0.05) prognostic factors in each of the final multivariable models

Analysis of virologic parameters by quartiles revealed that increasing viral load was consistently associated with increased all-cause mortality risk (Fig. 3). Fewer days with AdV viremia < 1000 copies/ml or days with undetectable AdV viremia showed a strong association with higher mortality risk. For the comparison of the quartile with the fewest days with viremia < 1000 copies/ml (Q1, ≤42 days) versus the greatest number of days (Q4, ≥105 days), the hazard ratio was 72.7 (95% CI 24.5–218.3). For undetectable viremia, the hazard ratio was 72.7 for patients with ≤21 days (Q1) of undetectable viremia versus those with ≥98 days (Q4) of undetectable viremia. AdV AAUC_0-16_ was also associated with all-cause mortality—patients in the highest quartile of AdV viral burden had an 11-fold increase in the hazard for mortality when compared with the lowest quartile (95% CI 5.3–23.6) (Fig. 3).

Of the children who died, 79% (34/43) had persistent AdV viremia at the last measurement. In those with AdV AAUC_0-16_ in the top quartile, 97% (30/31) of the patients who died still had AdV viremia at the last measurement. Corresponding analysis of nonrelapse-related mortality led to similar findings (Supplementary Figure 1).

**Fig. 4:**
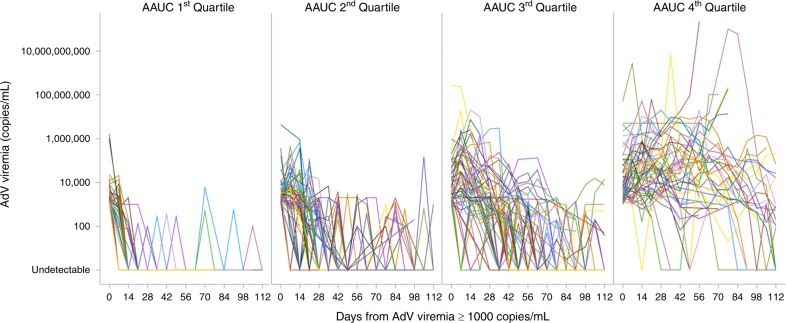
Both AdV peak and persistence contribute to total AdV viral burden (as measured by AdV AAUC)

**Fig. 5:**
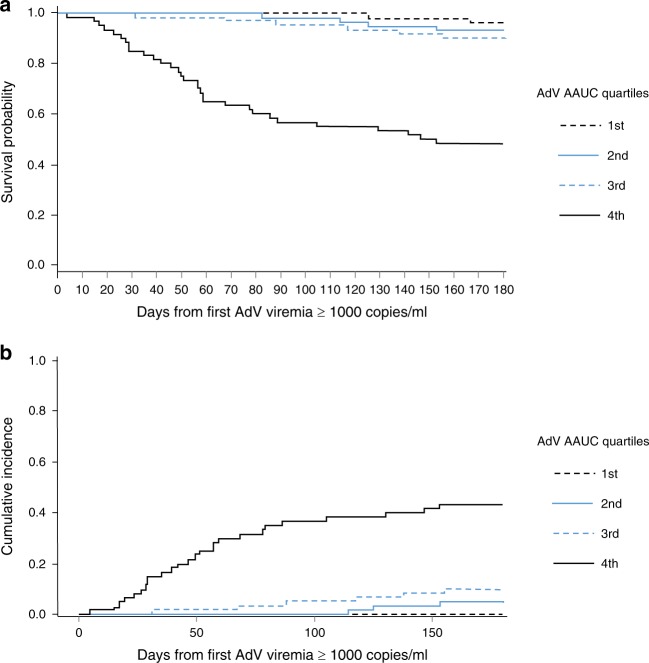
Higher AdV viral burden is associated with lower probability of 6-month overall survival. **a** Kaplan–Meier plot of all-cause mortality. **b** Cumulative incidence plot of nonrelapse-related mortality

Analysis of individual AdV viral load plots over time shows that peak and persistence of AdV viremia were far greater in the highest AdV AAUC_0-16_ quartile compared with the lowest quartile (Fig. 4). All-cause mortality was significantly greater in the highest quartile versus the lowest quartile (52%, 31/60 vs 3%, 2/60) (Fig. 5a), and the differences between quartiles were statistically significant (*P* < 0.0001 for all quartile comparisons from chi-square test).

Nonrelapse-related mortality was also greatest in the highest AdV AAUC_0-16_ quartile (43%, 26/60) compared with the lowest AdV AAUC_0-16_ quartile (0%, 0/60) (Fig. 5b). Differences between quartiles of AdV AAUC_0-16_ were statistically significant (*P* *<* 0.0001 for all comparisons from chi-square test).

## Discussion

AdVance is the first large retrospective multinational/multicenter study to examine the impact of AdV viremia on clinical outcomes in pediatric allo-HCT recipients. Whereas previous analyses have been from small or single-center studies [1, 14, 15], AdVance is a broad representative study incorporating data from 50 centers in seven countries across Europe, at least 37 of which treated one or more pediatric patients.

Different measures can be used to assess viral dynamics: peak viral load, the duration of infection, and the change in viral load over time. For cytolytic viral infections such as AdV, plasma viremia may provide a quantitative measure of tissue damage in the most commonly affected organs: the gastrointestinal tract, lungs, liver, or kidneys [17]. In our study, over half the pediatric allo-HCT recipients who developed AdV viremia surpassed the level of ≥1000 copies/ml, an accepted threshold for the initiation of pre-emptive treatment [7, 18]. Analyses of AdV viral load in these patients revealed a strong association with risk of mortality. Findings were similar for overall mortality and for nonrelapse-related mortality.

A lower number of days with undetectable AdV viremia or AdV viremia below the threshold of 1000 copies/ml (i.e., more prolonged AdV viremia) was a strong predictor of mortality. AdV viral burden, assessed by AdV AAUC_0-16_, was strongly associated with mortality, and pediatric allo-HCT recipients with the greatest viral burden (i.e., highest AdV AAUC_0-16_ quartile) were at substantially greater risk of death than those with the lowest viral burden (ie, lowest AdV AAUC_0-16_ quartile).

In separate multivariable models, all six AdV virologic measures were found to be an independent risk factor for mortality, along with previously identified risk factors for AdV-associated mortality, such as lymphocyte recovery or GvHD [19–21]. There was a high degree of concordance between the multivariable models, suggesting that both peak viremia and duration of infection were correlated with mortality.

Although competing risks for nonrelapse-related mortality (such as regimen-related toxicity, other infections, and GvHD) complicate attribution of cause of death to any one factor, the close temporal relationship of AdV viremia with death and the correlation of high viral burdens with mortality strongly suggest that AdV infection was a causative factor in most of these cases. Most of these deaths occurred within 2 months of first detection of AdV viremia ≥ 1000 copies/ml despite receiving standard of care; the vast majority of subjects who died still had AdV viremia at the time of death, also suggesting that AdV was likely causal in many of these cases.

While measures of viral dynamics—such as peak AdV viremia, change in viral load over time, or days with undetectable AdV viremia—are clinically relevant measures of AdV viremia, AdV AAUC appeared to be a sensitive measure of the total viral burden experienced by the patient, incorporating both the peak and persistence of AdV in the plasma. AUC has been used to assess the efficacy of antivirals against influenza A and respiratory syncytial virus in clinical trials [22, 23]. This parameter has also been used to assess the efficacy of treatments for HIV-1 [24]. Time-averaged AUC extends upon this measure by accounting for variability in follow-up due to the competing mortality risk.

The failure to respond to standard-of-care antiviral therapy was associated with increased mortality in this study. Our findings suggest that prompt and sustained control of AdV viremia with more potent antivirals is likely to have a substantial impact on mortality in this patient population.

In line with all observational studies, the findings from AdVance are subject to limitations due to the retrospective nature of data capture and the potential for bias that cannot be fully accounted for in the statistical models. There was no central laboratory or centrally driven treatment approach, and thus the differences in the threshold for initiating antiviral therapy and/or the AdV polymerase chain reaction assay from institution to institution reduces precision. Notably, however, a survey of practice patterns at the centers participating in the AdVance study showed that monitoring and treatment practices were broadly similar and in line with the guidelines of the European Conference on Infections in Leukemia [11, 18].

Currently there are no approved treatment options for AdV viremia in allo-HCT recipients, and IV cidofovir is commonly used off-label [25]. While IV cidofovir can help stabilize AdV viremia during the period preceding immune reconstitution, AdV clearance by cidofovir generally requires support from cellular immunity, and nephrotoxicity remains a substantial drug-related side effect limiting its use [13, 15, 16, 26, 27]. Clearly, there is a need for more effective and less toxic treatment options for these patients to improve clinical outcomes.

In summary, the AdVance study represents the largest and most robust evaluation of the risk for mortality associated with AdV viremia in pediatric allo-HCT recipients. These results are based on an observational ‘real-world’ clinical course of AdV infection in pediatric allo-HCT recipients under standard of care. Several measures of AdV viral load were strongly and independently associated with mortality after pediatric allo-HCT—both the peak and persistence of AdV viremia contribute to mortality risk. AdV AAUC_0-16_ incorporates both measures, with each log_10_ increase in AdV AAUC_0-16_ associated with a near-doubling of mortality risk; these data suggest that interventions able to reduce AdV viral burden should decrease mortality. Our findings show that AdV viral dynamic measures including AdV AAUC_0-16_ are useful indicators of outcome in pediatric allo-HCT recipients with AdV viremia. These markers may be usefully employed in clinical trials aimed at validating the efficacy of novel therapies that have the potential to reduce the risk of mortality in these patients.

## Supplementary information

Supplementary material
