# Supplementary material for: Association between adenovirus viral load and mortality in pediatric allo-HCT recipients: the multinational AdVance study
Source: Bone Marrow Transplant. 2019 Feb 25;54(10):1632–42. doi: 10.1038/s41409-019-0483-7 (PMC6957460; doi:10.1038/s41409-019-0483-7)

**Supplementary Table 1. Comorbidities**

| Arrhythmia: Atrial fibrillation or flutter, sick sinus syndrome, or ventricular arrhythmias |
| --- |
| Cardiac: Coronary artery disease, congestive heart failure, myocardial infarction, or ejection fraction ≤50% |
| Heart valve disease: Except mitral valve prolapse |
| Cerebrovascular disease: Transient ischemic attack or cerebrovascular accident |
| Moderate pulmonary: D_LCO_ and/or FEV_1_ 66%-80% or dyspnea on slight activity |
| Severe pulmonary: D_LCO_ and/or FEV_1_ ≤65% or dyspnea at rest or requiring oxygen |
| Peptic ulcer: Requiring treatment |
| Inflammatory bowel disease: Crohn disease or ulcerative colitis |
| Diabetes: Requiring treatment with insulin or oral hypoglycemics but not diet alone |
| Hepatic, mild: Chronic hepatitis, bilirubin > ULN to 1.5 × ULN, or AST/ALT > ULN to 2.5 × ULN |
| Hepatic, moderate/severe: Liver cirrhosis, bilirubin > 1.5 × ULN, or AST/ALT > 2.5 × ULN |
| Moderate/severe renal: Serum creatinine > 2 mg/dl, on dialysis, or prior renal transplantation |
| Rheumatologic: Systemic lupus erythematosus, rheumatoid arthritis, polymyositis, mixed connective tissue disease, or polymyalgia rheumatica |
| Psychiatric disturbance: Depression or anxiety requiring psychiatric consult or treatment |
| Prior solid tumor: Treated at any time point in the patient’s past history, excluding nonmelanoma skin cancer |

ALT, alanine transaminase; AST, aspartate aminotransferase; D_LCO_, diffusing capacity for carbon monoxide; FEV_1_, forced expiratory volume in 1 second; ULN, upper limit of normal.

**Supplementary Figure 1. Higher AdV viral load and burden were associated with a significantly greater risk of non relapse-related mortality.** AdV AAUC_0-16_, log_10_ of the time-averaged area under the AdV viremia curve over the 16 weeks following first AdV viremia ≥ 1000 copies/ml; Peak AdV viremia_0-16_, peak log_10_ AdV viremia over the 16 weeks following first AdV viremia ≥ 1000 copies/ml; Days with AdV viremia < 1000 copies/ml, number of days where AdV viremia was < 1000 copies/ml over the 16 weeks following first AdV viremia ≥ 1000 copies/ml; Days with undetectable AdV viremia, number of days where AdV viremia was undetectable over the 16 weeks following first AdV viremia ≥ 1000 copies/ml; 2-week change in AdV viremia, change in log_10_ AdV viremia in the first 2 weeks from first AdV viremia ≥ 1000 copies/ml. Q, quartile; ref, reference group. Note: Factors with *P* ≤ 0.20 in the univariate analysis were brought forward into the multivariable models, followed by a backward selection to keep the factors with *P* ≤ 0.10 in the final models. Lymphocyte count, sex, and renal replacement therapy were also significant (*P* < 0.05) prognostic factors in each of the final multivariable models.


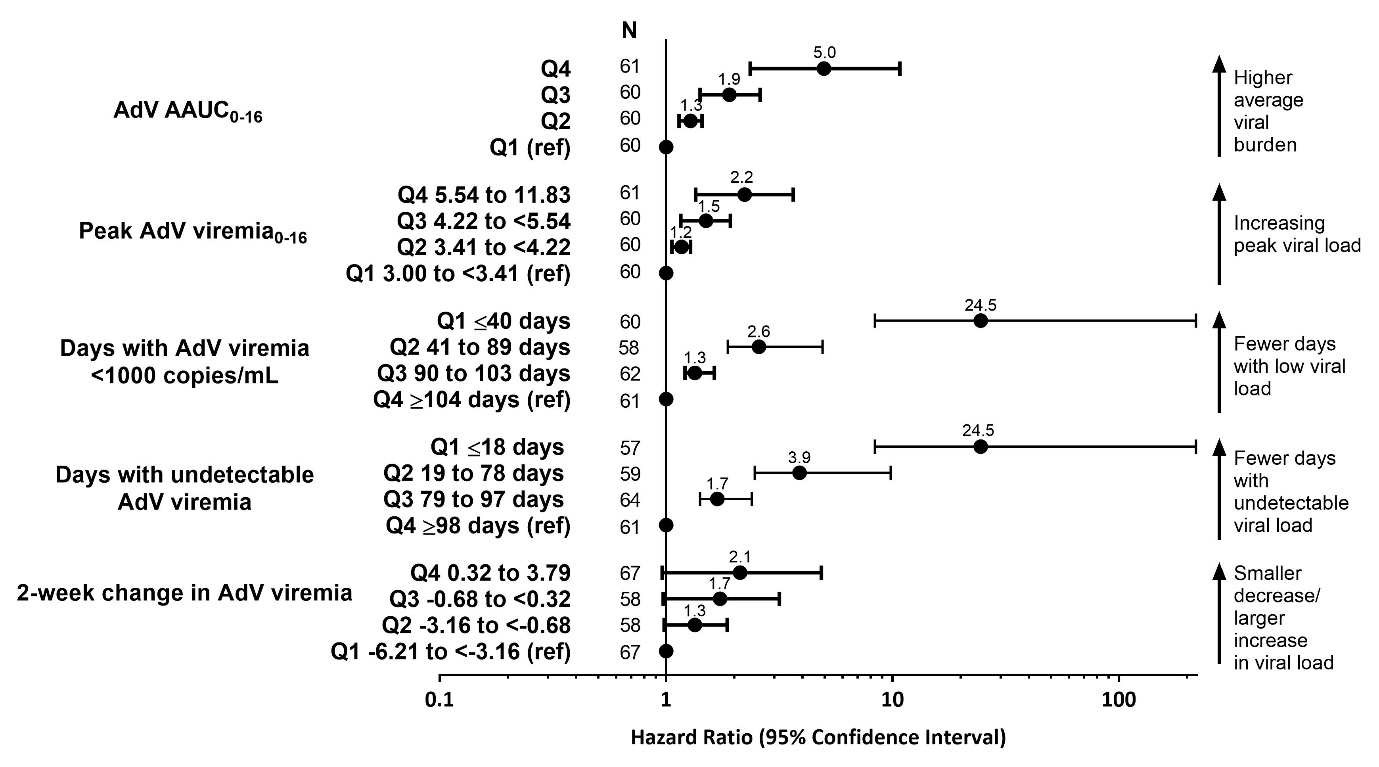

Supplement: Supplementary file 1 — Supplementary material [file 41409_2019_483_MOESM1_ESM.docx]
